# Easy Surface Functionalization and Bioconjugation of Peptides as Capture Agents of a Microfluidic Biosensing Platform for Multiplex Assay in Serum

**DOI:** 10.1021/acs.bioconjchem.1c00146

**Published:** 2021-06-11

**Authors:** Concetta Di Natale, Edmondo Battista, Vincenzo Lettera, Narayana Reddy, Gabriele Pitingolo, Raffaele Vecchione, Filippo Causa, Paolo Antonio Netti

**Affiliations:** † Center for Advanced Biomaterials for Healthcare@CRIB, Istituto Italiano di Tecnologia (IIT), Largo Barsanti e Matteucci 53, 80125 Naples, Italy; ‡ InterdisciplinaryResearch Centre on Biomaterials (CRIB), Università degli Studi di Napoli “Federico II”, Piazzale Tecchio 80, 80125 Naples, Italy; § Dipartimento di Ingegneria Chimica del Materiali e della Produzione Industriale (DICMAPI), University “Federico II”, Piazzale Tecchio 80, 80125 Naples, Italy; ∥ Biopox srl, Viale Maria Bakunin 12, 80125 Naples, Italy

## Abstract

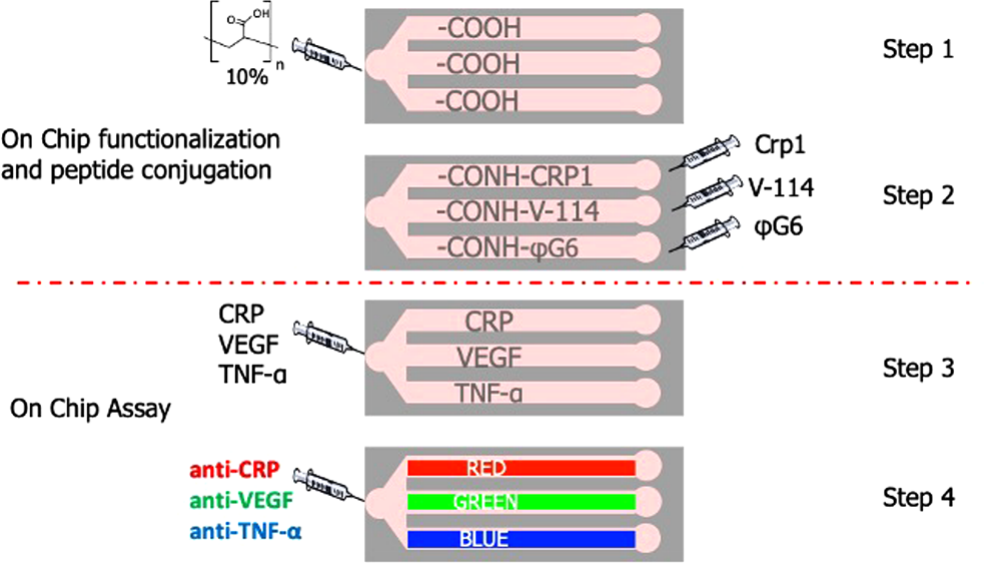

The development of assays for protein biomarkers in complex matrices is a demanding task that still needs implementation of new approaches. Antibodies as capture agents have been largely used in bioassays but their low stability, low-efficiency production, and cross-reactivity in multiplex approaches impairs their larger applications. Instead, synthetic peptides, even with higher stability and easily adapted amino acid sequences, still remain largely unexplored in this field. Here, we provide a proof-of-concept of a microfluidic device for direct detection of biomarker overexpression. The multichannel microfluidic polydimethylsiloxane (PDMS) device was first derivatized with PAA (poly(acrylic acid)) solution. CRP-1, VEGF-114, and ΦG6 peptides were preliminarily tested to respectively bind the biomarkers, C-reactive protein (CRP), vascular endothelial growth factor (VEGF), and tumor necrosis factor-alpha (TNF-α). Each PDMS microchannel was then respectively bioconjugated with a specific peptide (CRP-1, VEGF-114, or ΦG6) to specifically capture CRP, VEGF, and TNF-α. With such microdevices, a fluorescence bioassay has been set up with sensitivity in the nanomolar range, both in buffered solution and in human serum. The proposed multiplex assay worked with a low amount of sample (25 μL) and detected biomarker overexpression (above nM concentration), representing a noninvasive and inexpensive screening platform.

## 1 Introduction

In the past decades, the development of microassays able to detect and simultaneously monitor the levels and activities of a large number of proteins is becoming one of the hot topics in the biotechnological field.^1^ Frequently, these devices are made of microfluidic channels through which the analytes are detected by specific molecular binding events upon functionalization of the surface.^2^ These systems known as *lab-on-a-chip* provide several advantages, such as reduction of processing time, solvent and sample consumption, as well as enhanced sensitivity.^3^ By contrast, they still need complex chemical procedures able to control the regioselectivity of the immobilization reaction, in order to achieve a precise alignment and controlled uniformity of the biomolecule density on the inner surface of the microfabricated channels. Moreover, the fabrication process requires preservation of the native conformation, function, or activity of the immobilized molecular determinant.^4^ The polymeric surface functionalization involves the formation of three main chemical groups such as hydroxyl, carboxyl, and amine, and, less frequently, more selective groups, such as thiol, aldehyde, phosphate, or silane. Each surface modification requires a high degree of control because generation of a few of reactive groups induces the unspecific adsorption on the unfunctionalized hydrophobic polymer surface, thus affecting the three-dimensional structure of the bounded molecular determinant that inactivates its biological proprieties. Conversely, the extensive presence of functional groups, formed through an excessive surface functionalization, can induce a steric hindrance between analytes, leading to molecule deactivation.^5^ In this frame, after surface activation, DNA, antibodies and aptamers are generally employed as the main capture agents in lab on chip systems.^6^ Unfortunately, the low efficiency production and high manufacturing costs, together with the low stability of these devices and the cross reactivity of different antibodies used in the same system for the detection of different epitopes at the same time, have greatly limited the growth of biosensor applications.^6−8^ An affordable alternative method consists in employing synthetic peptides as capture agents. Peptides can be produced through an economically affordable methodology and purified in large quantities, with an efficient quality control;^9−13^ they also possess good stability that does not need particular environmental conditions such as temperature/pH variations, presence/absence of water molecules, or protease/nuclease degradation.^10^ Moreover, they can be isolated from combinatorial libraries and are able to bind target proteins with high affinity.^10,14,15^ In addition, thanks to the possibility to easily change/design their native amino acid sequence, they can be synthesized with a common sequence able to bind to the activated surface in order to avoid cross-reactivity phenomena in multiplex assays or to generate a more uniform deposition.^16^

Based on these statements, we selected three different peptides as model ligands of various inflammatory-cancer biomarkers: tumor necrosis factor-α (TNF-α), vascular endothelial growth factor (VEGF), and C-reactive protein (CRP). These sequences have been previously screened by surface plasmon resonance (SPR), phage display, and FACS methodologies, showing a high affinity for designated biomarkers.^17−19^ The area of interest of these biomarkers spreads through several inflammatory cancer-related diseases.^20−22^ In particular, several works have reported that VEGF, TNF-α, and CRP together with other important peripheral markers of inflammation such as interleukin (IL)-6, IL-1, IL-8, chemokines, and metalloproteinases (MMPs) are crucial in the onset of different kinds of cancer.^23−27^ For example, very high levels of VEGF, CRP, IL-6, and TNF-α (above nM concertation) were detected in glioblastoma patients with malignant prognosis, and in particular, their association is site specific in lung or colorectal cancer.^28,29^ Epidemiological studies showed that the inflammatory microenvironment, composed of a variety of cytokines, chemokines, and enzymes, induces the activation of oncogene related transcription factors, unleashes the production of tumor-promoting cytokines that in turn recruit and activate inflammatory cells. This mechanism leads to cell proliferation, migration, survival, and angiogenesis, increasing the risk of developing several kinds of cancer.^29^ In this frame, inflammatory biomarkers can be used to diagnose the presence of tumor and reveal the progression of the disease when such concentrations largely exceed a threshold level (around nM).^30^

**Figure 1:**
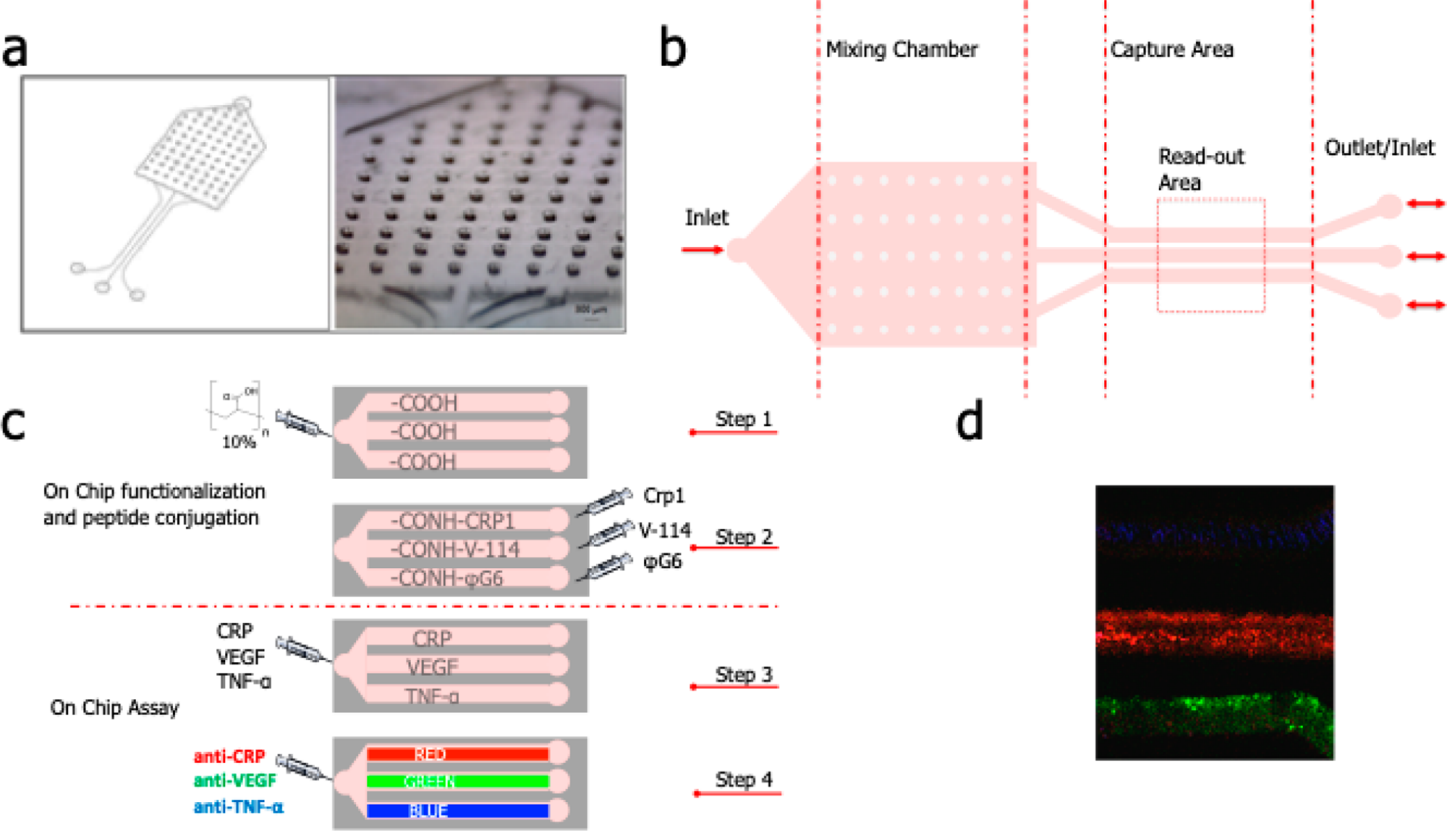
(a) Sketch and close-up by optical image of the negative master mold in PMMA. (b) Scheme of the chip with the evidence of the different regions. (c) Chip preparation and assay setup. (d) Readout area taken by confocal fluorescence microscope.

Recently, several approaches based on peptides as capture agents have been developed for the quantification of proteins in different biosamples such as liquor, plasma, saliva, or urine.^31,32^ However, these assays have often been coupled with very expensive analytical techniques as liquid chromatography with mass spectrometry detection (LC-MRM/MS).^33^ Here, we propose to use peptides as specific binding agents inside microchannels of a miniaturized device to realize multiplex detection for three cancer-related biomarkers. The miniaturized device (Figure 1) allows for easy functionalization with poly(acrylic acid) brushes (PAA) of the PDMS and a subsequent selective bioconjugation of the peptides in the capture area (Figure 1b). The assay setup is based on conventional design for immunofluorescence and quantified through microscopy.^34^

Thanks to the alternate loading approach, in single steps, we were able to perform more chemical reactions (Figure 1). The fabricated device was, indeed, tested in buffer and human serum in order to verify the capability to simultaneously reveal the presence of three different biomarkers in the nanomolar order, compatible with the physiological level of VEGF, CRP, and TNF-α in plasma. This level of detection could be enough to provide information above a threshold when multiple biomarkers are detected at time. Although in the literature the use of materials having antibody-grafted surfaces is usually far more effective to fabricate a diagnostically sensitive device for specific biomarker detection, the high production costs and the tricky storage conditions related to these biomolecules often limit their applicability.^29^

To fulfill the promise of emerging diagnostics, we report the fabrication and the performance of a surface-functionalized microdevice based on ligand peptide grafting, as an effective technology for diagnostic applications in the life science field.

To the best of our knowledge, few examples of microfluidic peptide-based biosensors are reported in the literature, and none of these can be compared with the robustness of the gold standard methods such as ELISA.^35,36^ Anyway, with an appropriate level of improvement for their user-friendly nature, they can have a major impact on clinical diagnostics creating a novel-based generation of biosensors for specific biomolecule targeting.^37^

## 2 Results and Discussion

### 2.1 Microfluidic Device

The microfluidic chip was fabricated by coupling micromilling and soft lithography technology based on polydimethylsiloxane (PDMS). PDMS is currently one of the most used materials for microfluidics chip fabrication, especially for its optical transparency.^38^ In addition, the fabrication of PDMS microchannels is particularly straightforward. They can be replicated from poly(methyl methacrylate) (PMMA) negative master molds (Figure 1a). Such a material can be easily machined by a low cost and fast prototyping technique such as micromilling.^39^ The PDMS patterned slab can then be easily sealed by oxygen plasma treatment. As shown in Figure 1b, the microfluidic device is composed by a mixing chamber with micropillars, to prevent the collapse of the soft PDMS large channel, a capture area realized by three parallel microchannels with independent outlets for the selective capture agents conjugation. The inlets will be alternatively used in the different phases (on-chip preparation and on-chip assay) (Figure 1c). The three parallel microchannels are set in order to create a readout area for the multiplex analysis and positioned at a distance to enter in the field of view of the fluorescence microscope (Figure 1d).

### 2.2 PDMS–PAA (Poly(acrylic acid)) Derivatization

**Figure 2:**
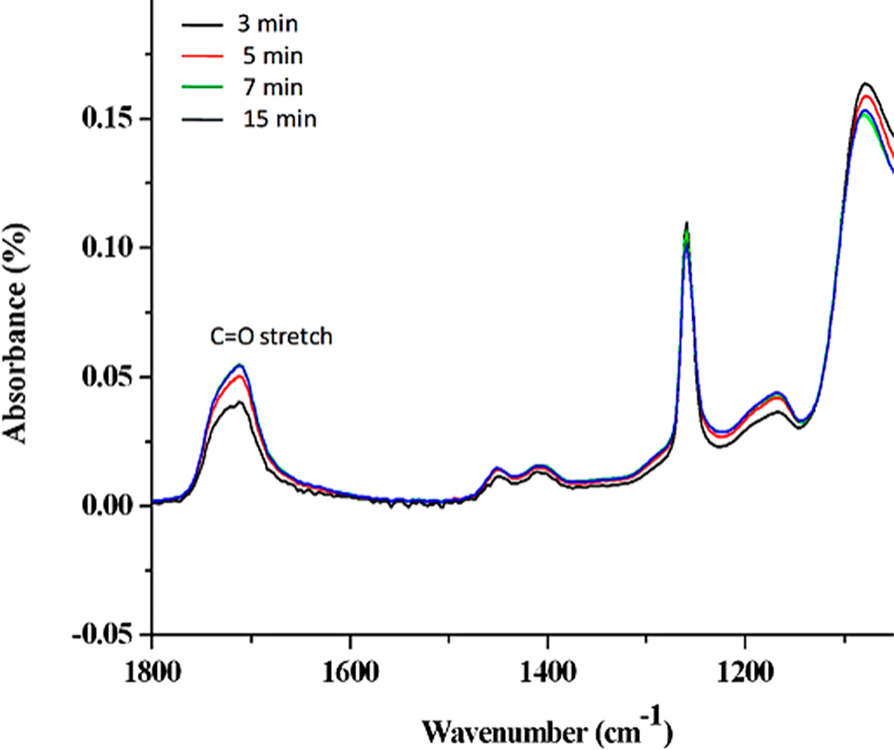
IR spectra of PDMS–PAA surfaces functionalized through reagent exposition at different times.

PAA brushes were grown on the PDMS surface previously activated with a 10% benzophenone solution in ethanol as described in the Experimental Section (Figure 1c) and according to the procedure already reported.^40^ The ideal PAA-derivatization at different UV-irradiation times was monitored by IR spectroscopy, analyzing the presence of the carboxyl C=O stretch at 1717 cm^–1^. In Figure 2, IR spectra of PDMS–PAA functionalized surface (10% of PAA–solution) at different UV-irradiation time periods, from 3 to 15 min, were reported. The enhancement of the acidic band at 1710 nm with the increasing time was evident; from 7 to 15 min, saturation was reached.

### 2.3 Peptide Grafting Optimization on PDMS–PAA Surface by IR and HPLC

**Figure 3:**
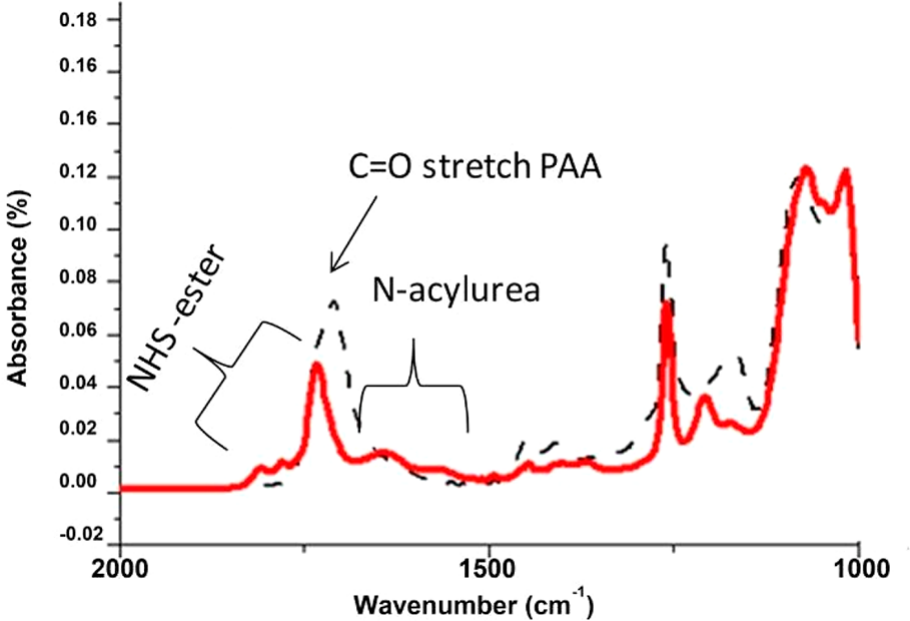
IR spectra of PDMS–PAA surface before (dashed line) and after activation treatment with EDC/NHS (0.1–0.2 M) (red line).

The peptide grafting was optimized with a model peptide (MP): Ac-βA-G-R-A-A-Y-A-K-NH_2_ as reported in the Experimental Section. Briefly, concentrations of peptide from 0.125 to 2 mg/mL were used to graft it onto the PDMS–PAA surface, previously activated with *N*-(3-(dimethylamino)propyl)-*N*′-ethylcarbodiimide hydrochloride (EDC)/*N*-hydroxysuccinimide (NHS) chemistry. IR spectra show the overlay of the PDMS–PAA surface before and after activation of PAA brushes with EDC/NHS mixture (Figure 3). The characteristic infrared bands at 1740, 1780, and 1815 cm^–1^ are related to the presence of the intermediate reaction product identified as NHS-ester, while the degradation product of the reaction, *N*-acylurea, is associated with doublet bands of amide at 1550 and 1650 cm^–1^ (Figure 3).

The amidation which occurred is shown in Figure S1. In particular, the presence of two bands at 1550 and 1670 cm^–1^, corresponding to amide I (peptide C=O stretch) and amide II (mostly peptide N=H bend) suggests the proper peptide conjugation on the surface (Figure S1A–C). At the same time, these bands are not shown on the non-preactivated surface, where the presence of two amide bands is very weak (Figure S1B–D) suggesting a simple adsorption. Moreover, in order to confirm the amidation of the PAA surface and to calculate the concentration of conjugated molecules, peptide solutions were analyzed before and after the grafting process by RP-HPLC, following the tyrosine signal at 275 nm (Figure S2). In more detail, the conjugated molecules were evaluated by measuring the peptide concentrations of unbound fractions after the grafting process. A saturation point on the treated surface (red curve) was reached at 1 mg/mL of peptide concentration. A nonlinear response was obtained for untreated surfaces where simple adsorption is the only way to stick peptide on the surfaces (black curve, Figure S3). In Tables S1 and S2, the amount of adsorbed molecules was reported for both EDC/NHS-treated and untreated surfaces.

### 2.4 *In Vitro* Affinity Assay between Peptides and Biomarkers

SPR experiments were performed by one-step injection with the aim of evaluating and confirming the binding constant between peptide and biomarkers. Analyte concentrations of 30 μM of VEFG-114 peptide and 245 μM of CRP-1 peptide were used. The one-step experiment was based on the Taylor dispersion theory, so a unique concentration of peptide was dispersed into a running buffer directly in the flow cell in order to have a final sigmoidal profile.^41,42^ By employing a 1:1 interaction model, a low micromolar dissociation value for peptides/biomarkers was shown in Figure S4A,B, and it is in agreement with the conventional SPR experiments reported in the literature.^17,19^ For the G6 peptide, the affinity against the TNFα protein was previously estimated by our research group in a recent study (data not shown).^18^

### 2.5 Inflammatory Biomarkers Detection through the Microfluidic Device

Surfaces of each microfluidic channel were grafted by one of CRP-1, VEGF-114, and G6 peptides; thus, any channel was exclusively specific for binding and detection of a single biomarker. Each peptide-grafted channel was tested by injecting PBS solution into the channel that contained different concentrations of the corresponding target. Biomarker sequestration was then revealed by immunofluorescent hybridization through injection of a mixed solution of primary fluorescent-labeled antibodies. The limit of detection (LOD) was determined by acquiring the images of the fluorescent surface by confocal microscopy, and the related fluorescent signals were quantified as reported by Jonkman and co-workers.^43^

**Figure 4:**
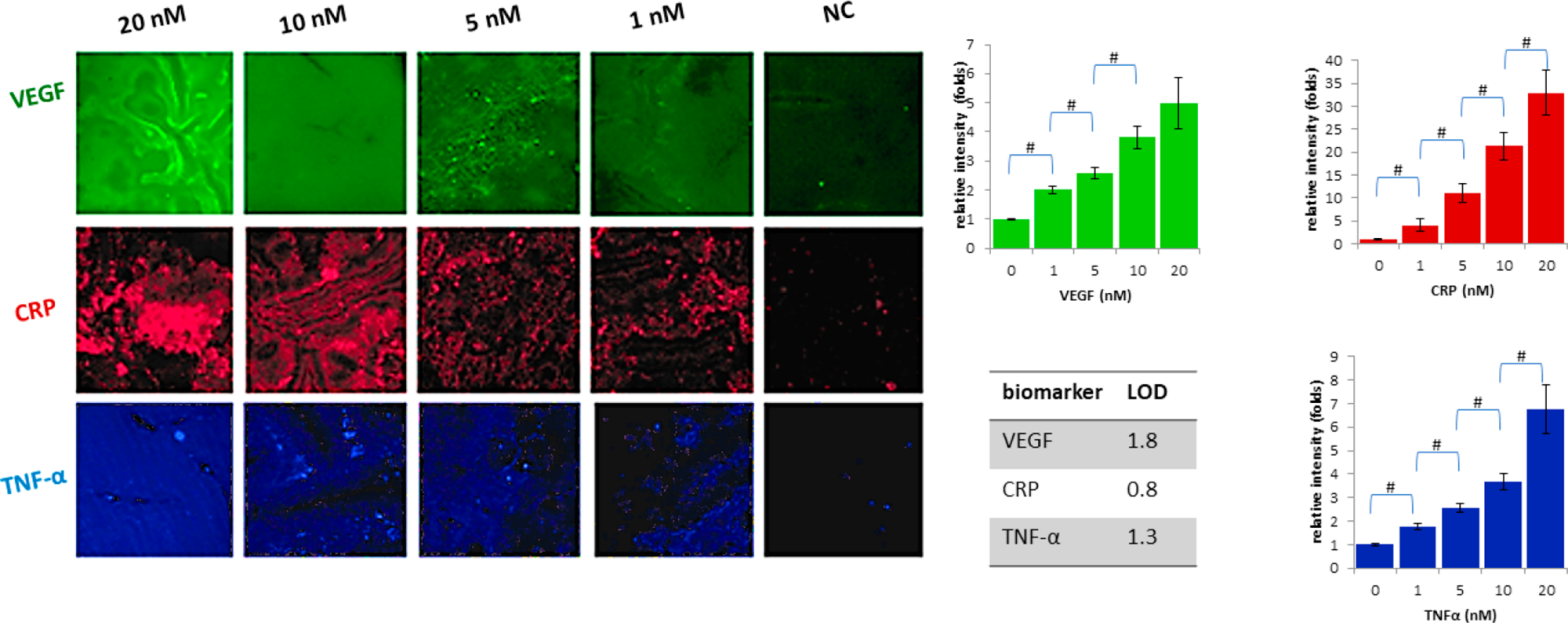
Fluorescence imaging of the channel surface after the capture of the biomarkers; on the right are bar graphs with significant difference (# *p* value <0.05). LOD determination for each target, as calculated from curves reported in SI Figure S5A–C.

As reported in Figure 4, each channel is able to specifically reveal the presence of biomarkers in solution at the nanomolar concentration (1.8 nM for VEGF, 0.8 nM for CRP, and 1.3 nM for TNF-α), compatible with the physiological range of VEGF, CRP, and TNF-α in plasma.^44−46^ In order to test suitability and specificity of the functionalized channels for multiplex analysis of inflammatory related biomarkers, the microfluidic system was furtherly tested by flushing 1 mL of PBS solution containing recombinant human VEGF, CRP, and TNF-α proteins at 5 nM final concentration as a concentration closer to the LOD and appreciable by our experimental setup. In this case, the immunodetection was performed by incubating each microchannel with a mixed solution containing fluorescent anti-VEGF, anti-CRP, and anti-TNF-α antibodies. The device was able to specifically detect the presence of each biomarker displaying the fluorescent signal only in the corresponding microchannel (Figure S6A–F). The specificity of the detection based on sequestrant peptides was verified through an analogous test on the microfluidic device whose channel surface was previously functionalized by a scramble peptide as negative control (Figure S7A).

**Figure 5:**
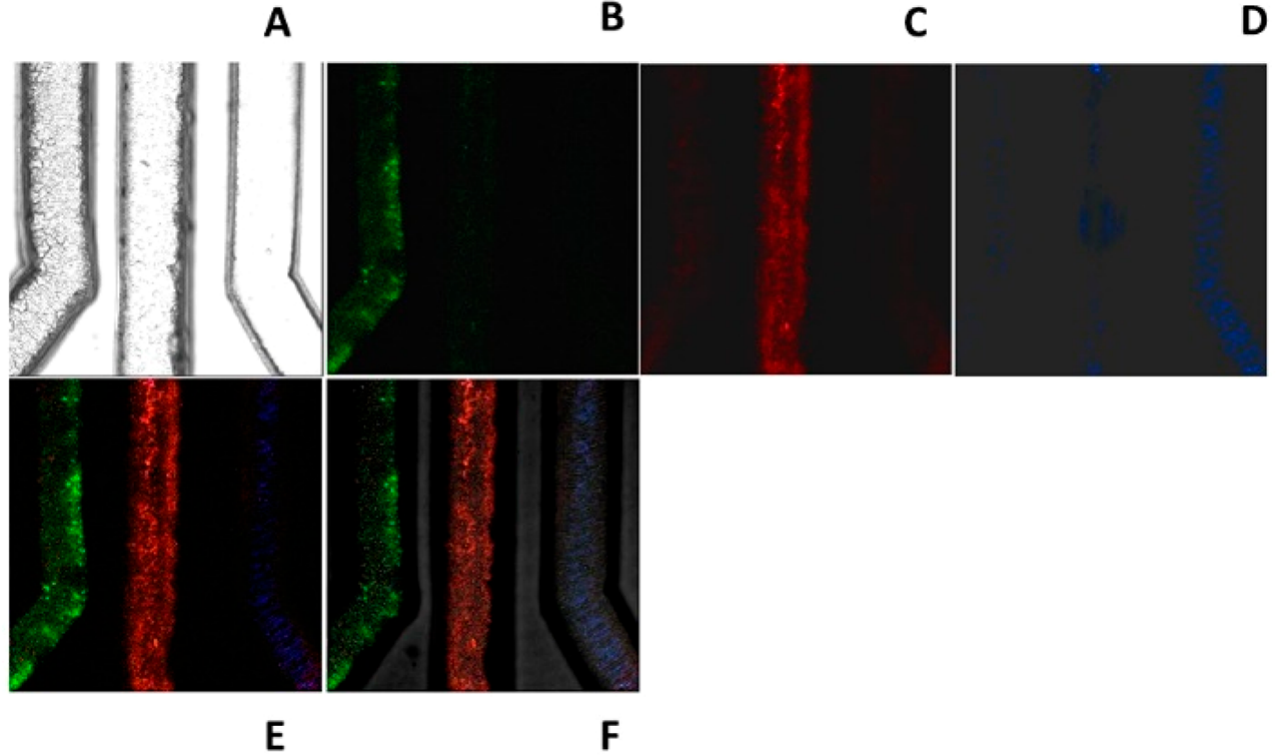
Detection of biomarkers spiked in human serum solution flushed in the functionalized microfluidic channel device. From left to right, the channels were functionalized with three different binding peptides: V114; CRP-1; φG6 (A). Immunofluorescence was performed with three different fluorescent antibodies: anti-VEGF (B), anti-CRP (C), and anti-TNF-α (D). Merging of the fluorescent signals (E). Overlay of the fluorescent channel with the transmission one (F).

Finally, we carried out an analogous assay by testing a human serum sample that contained 5 nM of each recombinant human EGF, CRP, and TNF-α biomarkers as the biological model (Figure 5A–F). The model sample was injected into the device, and it was treated through an immunofluorescent assay as previously reported for the PBS solution samples. The fluorescent image analysis reveals the presence of each biomarker into the serum exclusively in the corresponding specific channel, thus proving that the presence of serum proteins does not invalidate the assay. The potential ambiguous signals derived from human serum adsorption with the channel materials that can favor autoflorescence phenomena or unspecific interaction with the fluorescent antibody was confirmed by testing the device on the human serum in the absence of biomarkers (Figure S7B). Moreover, the presence of serum did not significantly interfere with the molecular capture of each biomarker, since the corresponding fluorescence signals were comparable to tho ones related to the PBS samples containing the molecular targets at the same concentration. As a matter of fact, the relative intensity of fluorescent signals related to the presence of VGF, CRP, and TNF-α was 2.2-fold, 8.8-fold, and 1.9-fold, respectively, even if there was a higher statistical uncertainty (standard deviation less than 30%).

However, the correspondence of the signals is strictly dependent on the resolution of the system, thus it is validated only in the function of the biological fluid that is currently processed and of the defined range of biomarker concentration.

Thanks to the obtained results and the benefits achieved such as alternate loading for multiple reactions, reduction of analysis time, use of a stable capture agent, achieving a better specificity and sensitivity, we spread out the potential applicability of our system in the multiplex analysis for diagnostic purposes.

## 3 Experimental Section

### 3.1 Materials

Reagents for peptide synthesis (Fmoc-protected amino acids, resins, activation, and deprotection reagents) were purchased from Iris Biotech GmbH (Waldershofer Str. 49–51, 95615); EDC/NHS, PDMS, and CRP (C- Reactive Protein) were from Sigma-Aldrich. VEGF (Recombinant Human VEGF165) was purchased from Peprotech. Anti-CRP (Anti-C Reactive Protein antibody (FITC) (ab19174)) and Anti-VEGF (Anti-Recombinant Human VEGF antibody (FITC)) were from Abcam. TNF-α and Anti-TNF-α (Anti-Tumor Necrosis Factor-α antibody (FITC)) were from Prospec. Solvents for peptide synthesis and HPLC analyses were purchased from Sigma-Aldrich; reversed-phase columns for peptide analysis and the LC–MS system were supplied, respectively, from Agilent Technologies and Waters (Milan, Italy). All SPR reagents and chips were purchased from AlfaTest (Rome, Italy). PMMA substrates used in this study were purchased from the same batch of the polymer supplier (Good Fellow Cambridge Limited, England); Fluorolink S10 was from Solvay. Pooled human serum from healthy donors was supplied by Lonza (Life Technology Ltd., Paisley, UK). All chemicals were used as received.

### 3.2 Fabrication of Microfluidic Device

The device consists of a chamber with an inlet and three parallel microchannels with one outlet each (Figure 1a,b). The chamber having an area of 67 mm^2^ is integrated with micropillars of 300 μm in diameter and depth. The square microchannels are 300 μm in depth (d) and width (w). The fabrication process consists of four steps: (1) preparation of the chip draft using Draft sight (a Cad Software); (2) micromachining of PMMA layers; (3) double PDMS replica; and (4) finally a bonding process via oxygen- plasma treatment. A micromilling machine (Minitech Machinery Corporation) was used to fabricate the PMMA master with the features of the final device. The certified positioning accuracy of the three axes are 12”/300 mm in *x*-axis, 9”/228 mm in *y*-axis, and 9”/228 mm in *z*-axis. To standardize the fabrication process, the PMMA substrates used in this study were purchased from the same batch of the polymer supplier (GoodFellow Cambridge Limited, England). The microtools used in the microfabrication process were two flute endmills of 300 and 889 μm in diameter (Performacemicrotool, USA). During the micromilling process, spindle speed, feed speed, and plunge rate per pass were set to 12,000 rpm, 15 mm s^–1^, and 20, respectively. After the preparation of a PMMA master with negative features, open square microchannels in PDMS were obtained by double replica molding onto the starting master (Figure 1a). PDMS replicas were fabricated from a mixture of PDMS precursors mixed in ratio 10:1 with the curing agent and by using a thermal curing protocol at 80 °C for 2 h. Particularly, in order to prevent adhesion of the negative PDMS replicas on the positive PDMS mold, the latter was treated with oxygen plasma to activate the surface using a plasma chamber (Plasma prep II, SPI) for 1 min at a pressure of 0.3 mbar and power of 37 W, and then immersed for about 2 min into a silane solution (i.e., a mixture of 94% v/v isopropanol, 1% acetic acid, 1% Fluorolink S10, and 4% deionized water) and placed in an oven at 75 °C for 1 h, allowing complete reaction of the master surface with the fluorinated polymer.^47^ To obtain the closed PDMS chip, we treated the final PDMS replica and a glass slide previously coated with a thin PDMS layer through oxygen plasma activation, using a plasma chamber (Plasma prep II, SPI) for 1 min at a pressure of 0.3 mbar and power of 37 W. The glass coating process involved depositing a small undiluted PDMS droplet (around 1 mL) onto the center of the glass and then spinning at high speed (2000 rpm for 20 s). The bonding was then finalized in a controlled environment (temperature 80 °C for 2 h).

After the bonding, the PDMS surface was modified by engrafting PAA following the procedure explained in the next section. All the liquids were injected from the primary inlet by using a syringe pump under microscope control (Figure 1c). Benzophenone 10% in acetone was injected and allowed to stand for 1 min, and then distilled water washings were performed by a syringe pump operated at 50 μL/min for at least 5 min. The engraftment was performed by filling the device with a 10% solution of acrylic acid and all the other additives and cured for 15 min by a UV lamp. Subsequently, the reaction solution was exchanged and the device washed several times with PBS solution and left overnight in the same solution, ready for the subsequent peptide conjugation step.

### 3.3 PDMS–PAA (Poly(acrylic acid)) Engraftment

Sacrificial PDMS samples were prepared according to the instructions of the manufacturer at a 1:10 curing agent/prepolymer ratio. The mixture was poured in a Petri dish and after degassing was cured in an oven at 80 °C for 1 h. The cured polymer was cut into pieces and poly(acrylic acid) was engrafted following the procedure described by Albritton et al.^36^ Briefly, the pieces were submerged for 1 min in an acetone 10% (W/V) benzophenone solution and then washed with deionized water 3 times. An aqueous solution of PAA (ranging from 3% to 10%), NaIO_4_ (0.5 mM), and benzyl alcohol (0.5% W/V) was dropped on the surfaces (10 μL/cm^2^) and covered with a rim glass.^47−52^ They were irradiated with a UV lamp at 360 nm (Black Ray 100 W, Ted Pella) for different time periods (from 1 min to 1 h), and after 3 washing steps, they were used for further conjugation. The same process of engraftment was applied to intact closed microchannels just by flowing all the reagents in the same way by syringe pumps.

### 3.4 Peptide Synthesis and Conjugation to PDMS–PAA Surfaces

Solid-phase syntheses of the CRP-1, VEGF-114, G6 peptides, and of the model peptide (Ac-βA-G-R-A-A-Y-A-K-NH_2_) (Table 1) were performed on a fully automated multichannel peptide synthesizer (Biotage Syro Wave). They were synthesized in the amidate version, employing the solid-phase method following standard Fmoc strategies as reported elsewhere.^53−55^ CRP-1 and VEGF-114 peptides were cyclized to obtain their active version. The cyclization process was performed using a concentration of 0.1 mg/mL of peptides dissolved in 10 mM phosphate buffer pH 7.4 for 2 days. The reaction that occurred was confirmed by LC–MS (Figures S8A,B and S9A,B). Products were purified by preparative RP-HPLC applying a linear gradient of 0.1% TFA CH_3_CN in 0.1% TFA water from 5% to 95% over 5 min at a flow rate of 5 mL/min.

**Table 1:**
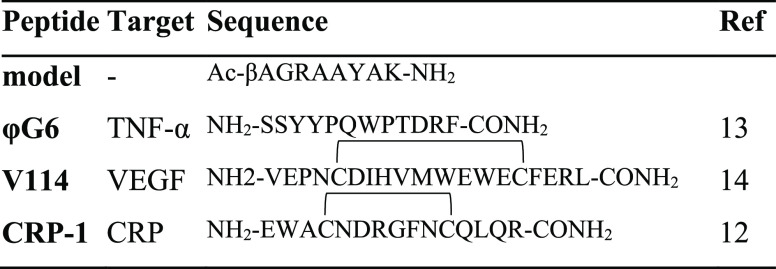
Synthesized Sequences

The model peptide Ac-βA-G-R-A-A-Y-A-K-NH_2_ was used for the optimization of the covalent conjugation to the PDMS–PAA by using EDC/NHS. The PAA surface was first treated with a 0.1 M EDC/0.2 M NHS mixture in water for 10 min,^56^ and afterward a solution of peptide (from 0 to 2 mg/mL) in carbonate buffer 10 mM pH 8.5 was added. The formation of the amide bond was monitored by IR analyzing the presence of peaks corresponding to amide I and amide II at 1660 and 1550 nm, respectively. In order to confirm the good amidation of the PAA surface and to calculate the amount of adsorbed moles, peptide solutions were analyzed before and after the PDMS–PAA grafting process by RP-HPLC, following the tyrosine signal at 275 nm.

### 3.5 Surface Plasmonic Resonance (SPR)

SPR experiments were performed by one-step injection. VEGF and CRP protein were immobilized at a concentration of 100 μg/mL in a 10 mM acetate buffer pH 4.5 and 3.5, respectively, (flow 10 μL/min, injection time 20 min) on a COOH1 SensiQ sensor chip, using EDC/NHS chemistry (0.4 M EDC/0.1 M NHS, flow 25 μL/min, injection time 4 min), achieving an 800 RU signal. Groups of reactive residues were deactivated by treatment with ethanolamine hydrochloride 1 M, pH 8.5. The reference channel was prepared by activation with EDC/NHS and deactivation with ethanolamine. Analyte concentrations of 30 μM for VEFG-114 peptide and 245 μM for CRP-1 peptide were used with a flow rate of 100 μL min^–1^ and a 300 s dissociation time. In this kind of experiment, the volume of sample was configured as the percentage of dispersion loop volume; thus, in order to have a longer plateau at full concentration, the largest percentage (100%) was used. As to bulk standard cycles, 3% of sucrose was used. For all experiments, kinetic parameters for both peptides were estimated assuming a 1:1 binding model and using QDAT software (SensiQ Technologies).

### 3.6 Setting of the Microfluidic Device for Biomarkers

Microfluidic device channels were functionalized for multiplex detection of biomarkers. V114, CRP-1, and φG6 peptides were immobilized filling any channel with a PBS solution containing 2 mg/mL of one peptide as well as previously described. The device was tested by flushing 1 mL of a testing solution containing different concentrations of VEGF, CRP, and TNF-α proteins in PBS. Functionalization through a scramble peptide (Ac-βA-G-R-A-A-Y-A-K-NH_2_) was used for negative controls. The resident time of the testing solution was 2 h. After protein incubation, the device was washed by 3 mL of PBS and incubated with a mixed solution of primary fluorescent anti-VEGF, anti-CRP, and anti-TNF-α antibody (dilution 1:10 in PBS) overnight. Fluorescence analysis was performed by a Leica SP5 confocal microscope. Bright field and fluorescence images using a HCX IRAPO L 25×/0.95 water objective were acquired. Images were acquired with a resolution of 1024 × 1024 pixels, zoom 1, and 2.33 A.U. Analogous experiments were performed by detecting biomarkers in human serum (1×), dissolved at a final concentration of 5 nM. Autoflorescence and the background signal were determined by fluorescence analysis of each channel surface incubated with PBS or human serum solution without biomarkers. All experiments were performed in triplicate at room temperature. Ten images of different areas of the surface were acquired for each sample.

### 3.7 Multiplex Immunofluorescence Assay: Selection of Protein- Binding Peptides for Specific Marker Detection and Quantification

Each peptide was immobilized on the PDMS–PAA surface in a specific channel as previously described. Different concentrations of pure human recombinant TNF-α (Abcam), VEGF (Abcam), and CRP (Sigma-Aldrich) proteins were solubilized in PBS or plasma and incubated by flushing 25 μL of biomarker solutions into the corresponding channel for 2 h. After incubation, the channels were washed 3 times with 3 mL of PBS. Biomarker detection was performed by immunofluorescent detection through primary anti-TNF-α, anti-VEGF, and anti-CRP antibody (Abcam), labeled with Alexa Fluor 488, Alexa Fluor 568, and Pacific Blue dye, respectively, by Molecular Probes Antibody Labeling Kits (ThermoFisher Scientific). Fluorescence analysis was performed by a Leica SP5 confocal microscope. Bright field and fluorescence images using a HCX IRAPO L 25×/0.95 water objective were acquired. Images were acquired with a resolution of 1024 × 1024 pixels, zoom 1, and 2.33 A.U. All experiments were performed at room temperature. The LOD range related to each was determined by image analysis through the ImageJ tool (https://imagej.nih.gov/ij/).^57^

### 3.8 Statistical Analysis

All the experiments were performed at least three times, reported as mean ± standard deviation, and were analyzed statistically by paired Student’s *t*-test. Significant difference was determined at *P* values smaller than 0.05.

## 4 Conclusions

Miniaturized devices for protein quantification represent one of the most promising fields in diagnostics. The main challenge for their successful commercialization is to combine an easy setup and the low-cost manufacturing with the high stability of the capture agents.^58^ To do this, the knowledge around surface capturing agent immobilization, biological matrix interferences, fluid device control, or signal detection techniques is necessary.^59^ Microfluidic devices use several methods for the capturing agent immobilization, but the most popular is the covalent binding of antibodies onto the surfaces.^7^ Unfortunately, these molecules show excessive production cost and low stability during the chemical grafting reaction, and these are among the reasons why the current miniaturized microfluidic systems remain in academic environments and are still disconnected from the industrial realities.^10,14,15^ To overpass these limitations, peptide nucleic acids (PNAs)^60^ and peptide ligands are becoming gradually popular as reagents for molecular biomarker recognition. In this area, researchers have used microfluidics to generate these novel affinity reagents to specifically bind nucleic acids targets,^61^ proteins,^62^ or small molecules.^63^ Novel miniaturized devices for plasma biomarkers should engage low-cost manufacturing processes involving simple materials and designs that are easily scalable without compromising the assay sensitivity.^64^ These inexpensive techniques have enabled researchers to apply microfluidics in the molecular point-of-care (POC) diagnostics area accounting for the main segment of the microfluidics market.^65^ In this context, our device is suitable, eliminating the complexity and the high cost of the microfluidic system, using no expensive materials such as PDMS and PMMA that are also able to be industrially scalable and employ peptides (chemically stable and economically produced molecules) as capturing agents. We demonstrated simultaneous detection of three different biomarkers in human serum with a level of quantification in the nanomolar range, enough to provide information on their overexpression above a threshold. Such information along with multiplex capability can give information on the pathological status and provide a useful tool for inexpensive screening. Moreover, with suitable adjustments to the setup we could reach comparable LOD of ELISA, but with smaller sample volume (<25 μL).

In conclusion, this miniaturized device integrates specific features with different levels of novelty: (a) easy in situ surface modification; (b) regioselective bioconjugation with capture molecules; (c) use of peptides as capture molecules; (d) multiplex analysis of cancer related biomarkers by using conventional immunofluorescence protocols. Thanks to these features, we can perform simultaneous multiple biochemical recognitions, reducing the reaction and analysis time and preserving the molecules from degradation phenomena. We think that our device could open the route for designing and planning future efficient and low-cost diagnostic tools to contribute to the early diagnosis of inflammatory related pathologies that often have a central role in the onset of different kinds of cancer.

## Supporting Information Available

IR spectra, HPLC and titration curves, One Step injection experiments, Images showing detection of biomarkers dissolved in PBS solution and controls, Mass spectra, Amount of adsorbed moles on EDC/NHS treated and untreated surfaces (PDF)
