# Supplementary material for: Easy Surface Functionalization and Bioconjugation of Peptides as Capture Agents of a Microfluidic Biosensing Platform for Multiplex Assay in Serum
Source: Bioconjug Chem. 2021 Jun 11;32(8):1593–601. doi: 10.1021/acs.bioconjchem.1c00146 (PMC8382222; doi:10.1021/acs.bioconjchem.1c00146)
Supplement: Supplementary file 1 — bc1c00146_si_001.pdf [file bc1c00146_si_001.pdf]

Easy surface functionalization and bioconjugation of peptides as capture agents of a microfluidic biosensing platform for multiplex assay in serum.

Concetta Di Natale[a,b,†], Edmondo Battista[a, b,†,*], Vincenzo Lettera[a, d], Narayana Reddy[a], Gabriele Pitingolo[a,e], Raffaele Vecchione [a], Filippo Causa[a,b, c,*] and Paolo Antonio Netti[a,b,c]

[a] Center for Advanced Biomaterials for Healthcare@CRIB, Istituto Italiano di Tecnologia (IIT), Largo Barsanti e Matteucci 53, 80125 Naples, Italy

[b] InterdisciplinaryResearch Centre on Biomaterials (CRIB), Università degli Studi di Napoli "Federico II", Piazzale Tecchio 80, 80125 Naples, Italy

[c] Dipartimento di Ingegneria Chimica del Materiali e della Produzione Industriale (DICMAPI), University “Federico II”, Piazzale Tecchio 80, 80125 Naples, Italy

[d] Biopox srl, Viale Maria Bakunin 12, 80125 Naples, Italy.

[e] Current affiliation, BIOASTER, Technology Research Institute, 28 rue du Docteur Roux, 75015 Paris, Ile-de-France, France

Corresponding Author

*E-mail: edmondo.battista@unina.it, causa@unina.it.

† These authors equally contributed.

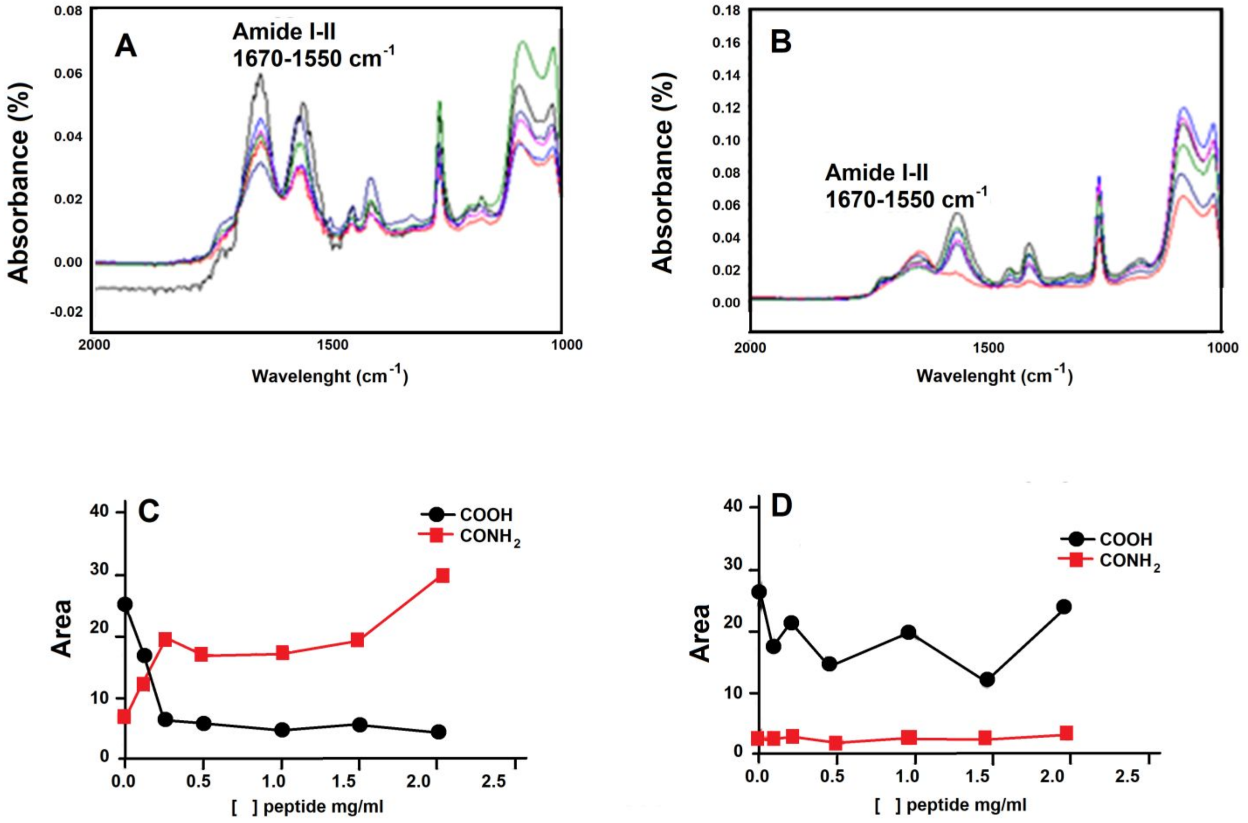

Figure S1. IR spectra of PDMS-PAA after peptide amidation. A) Whole IR spectra of amidation reaction after EDC/NHS reaction at different concentrations of peptide from 0.125mg/mL to 2mg/mL. B) Surface without EDC/NHS treatment. C-D) Quantification of the area of the amide and acid bands according to the peptide concentrations.

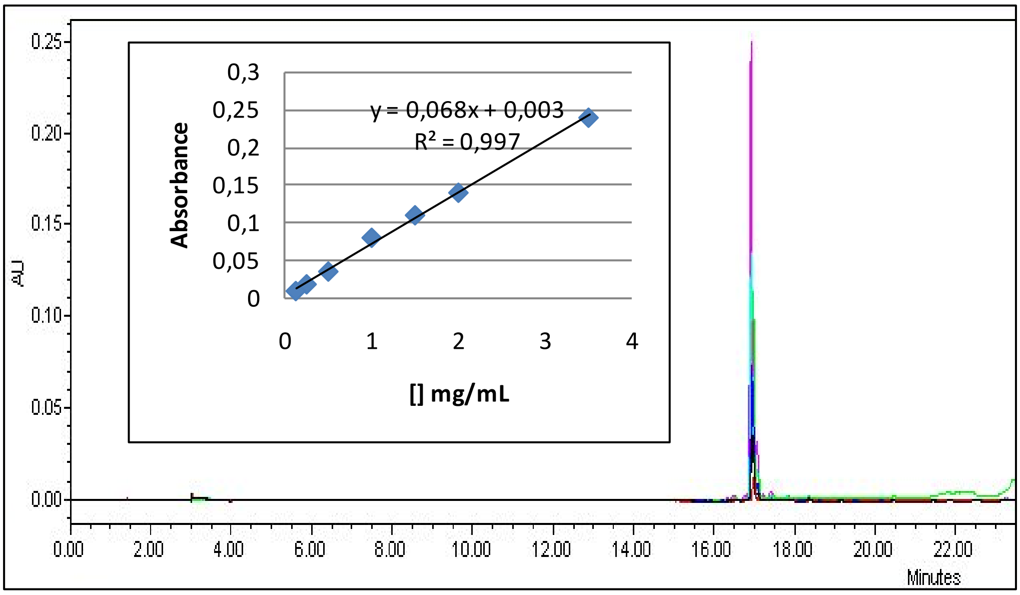

Figure S2. HPLC- titration curve of model peptide at 275 nm from 0 to 4 mg/mL.

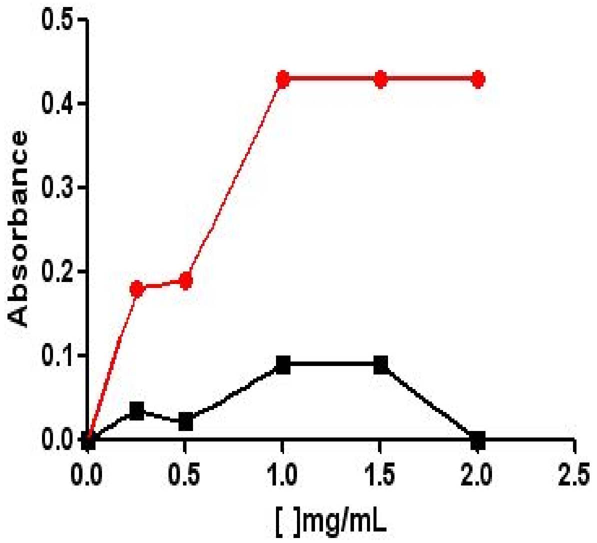

Figure S3. UV-vis signal at 275 nm of bound fractions corresponding to different concentrations of peptide for both surfaces. Red curve (pre-activated surface), black curve (not activated).

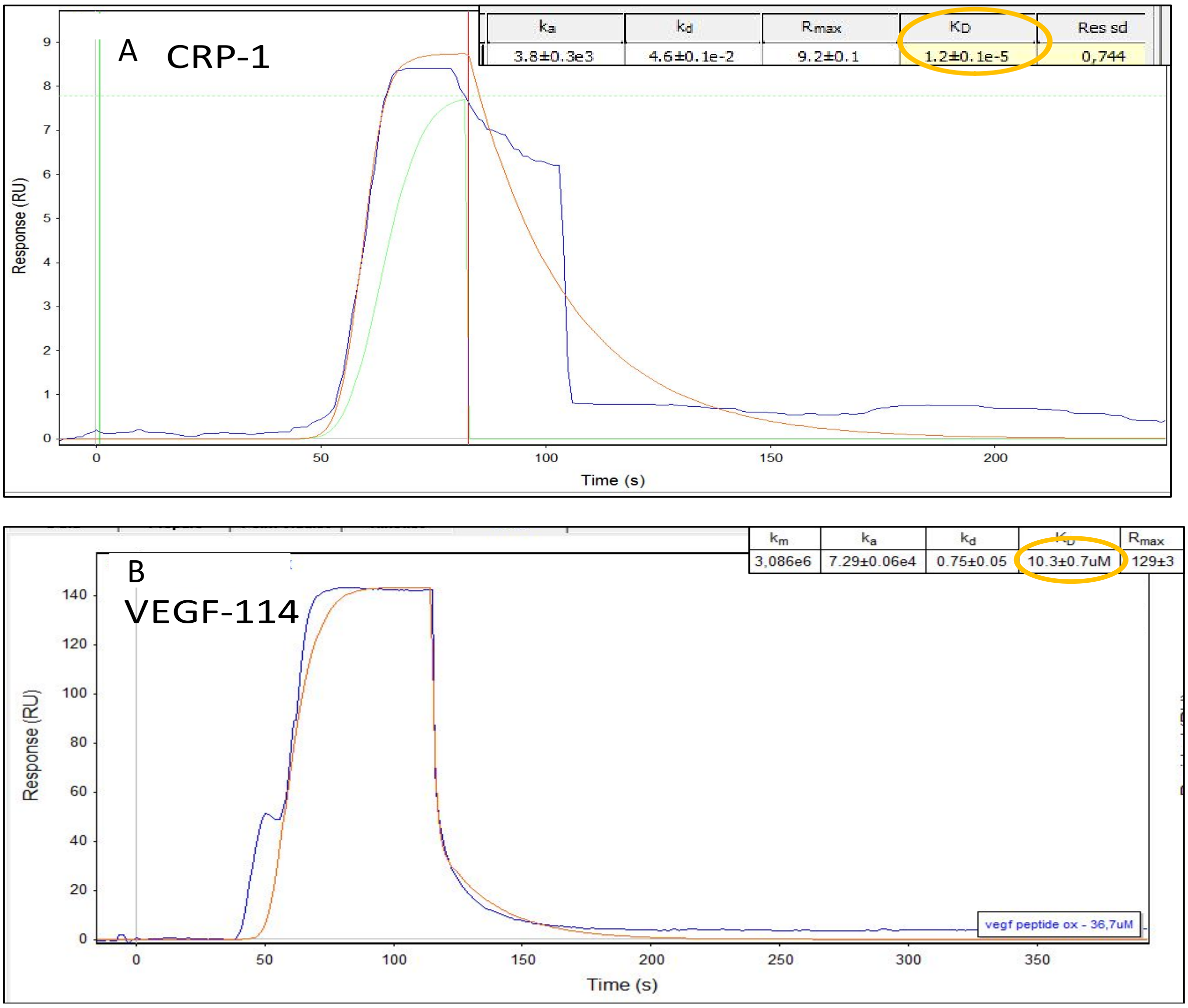

Figure S4. One Step injection experiments. A) Binding of CRP-1 (245µM) and CRP protein. The evaluated KD was 12±0.1 µM. B) Binding of VEGF-114 (30µM) and VEGF protein with a KD of 10 ±0.7µM.

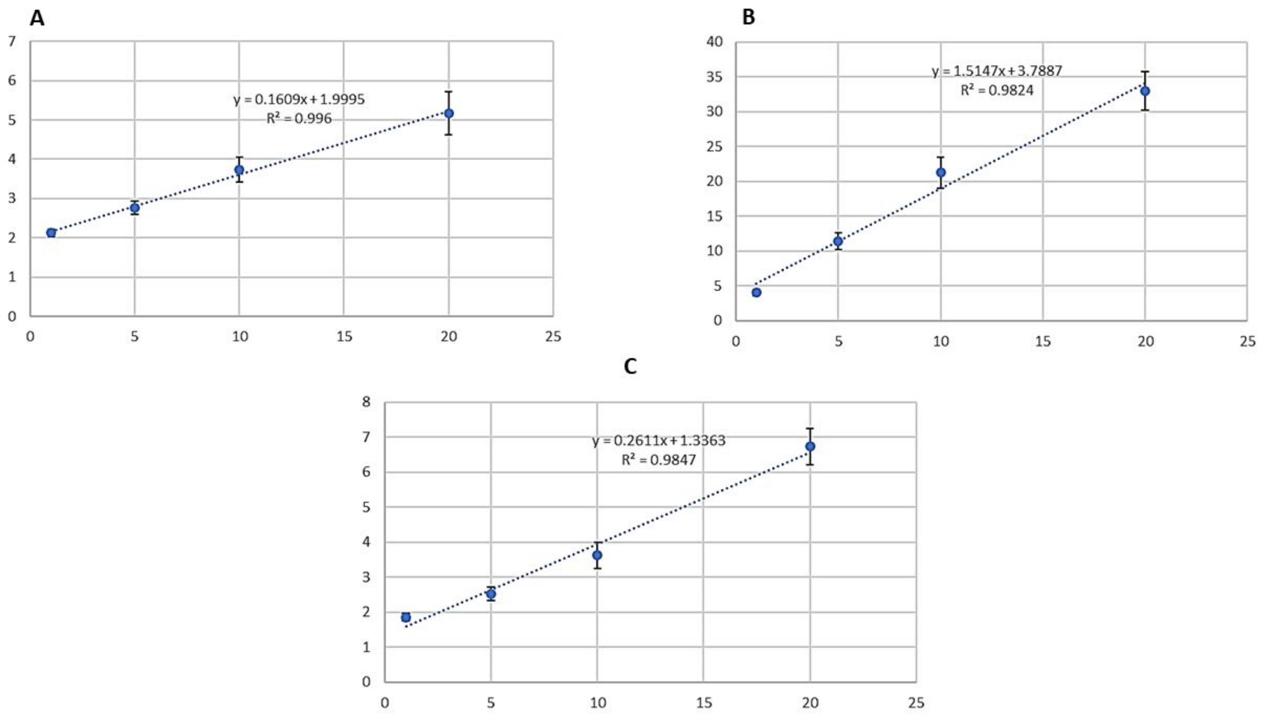

Figure S5: Titration curves of A) VEGF, B) CRP, C) TNFα used for LOD calculation.

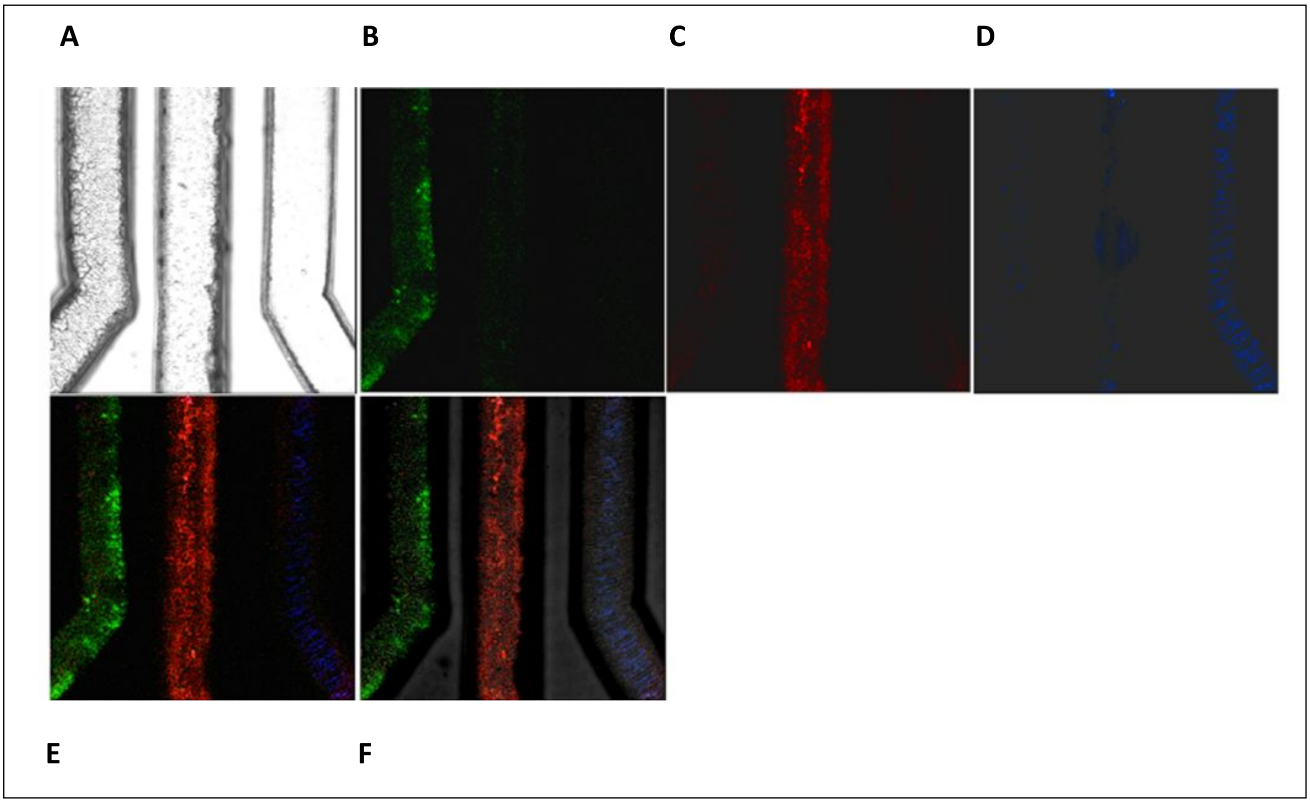

Figure S6. Detection of biomarkers dissolved in PBS solution flushed in functionalized microfluidic channel device. From left to right the channels were functionalized with three different binding peptides as follow: V114; CRP-1; ϕG6 (A). Immunofluorescence was performed with three different fluorescent antibodies: anti-VEGF (B), anti-CRP (C), anti-TNF-α (D). E-F) Merge of all three channels and their overlay with transmission one, respectively.

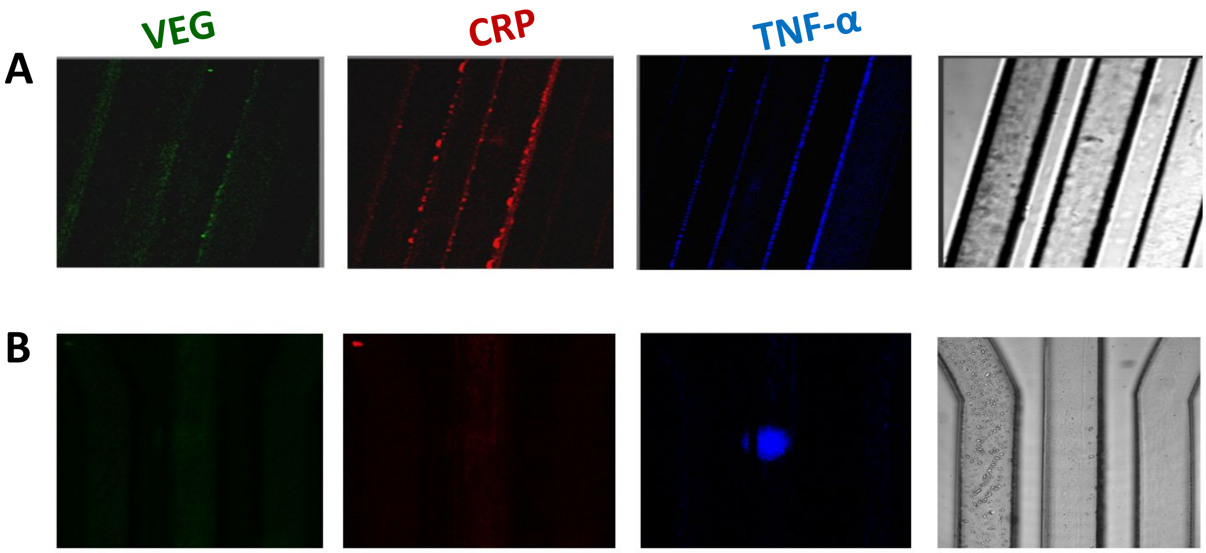

Figure S7: negative controls of microfluidic channels. A) Detection of biomarkers dissolved in PBS solution flushed in functionalized microfluidic channel device. Each channel was functionalized with the scramble peptide. From left to right the immunofluorescence was performed with three different fluorescent antibodies: anti-VEGF, anti-CRP, anti-TNF-α. **B)** Human serum without biomarkers was processed on the fabricated microfluidic device, whose channels were functionalized with three different binding peptides from left to right images as follow: V114; CRP-1; ϕG6. Immunofluorescence was performed with three different fluorescent antibodies: anti-VEGF, anti-CRP, anti-TNF-α. The transmission channel is also reported on the last image on the right for each device.

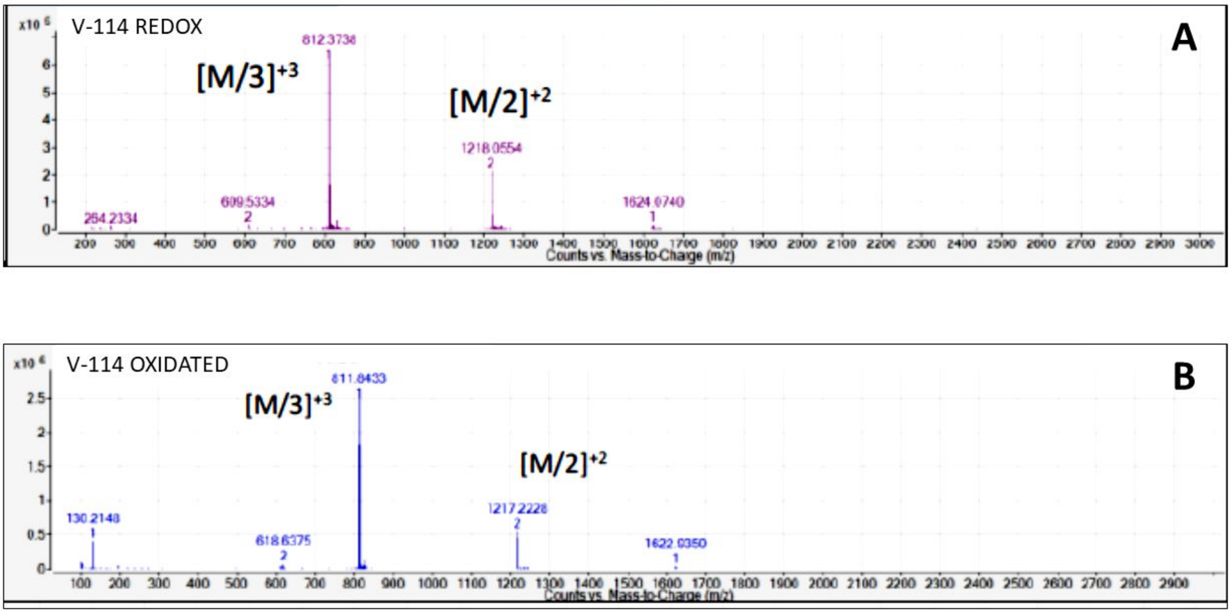

Figure S8: Mass spectrum of V-114 peptide: A) reduced version, B) oxidated compound.

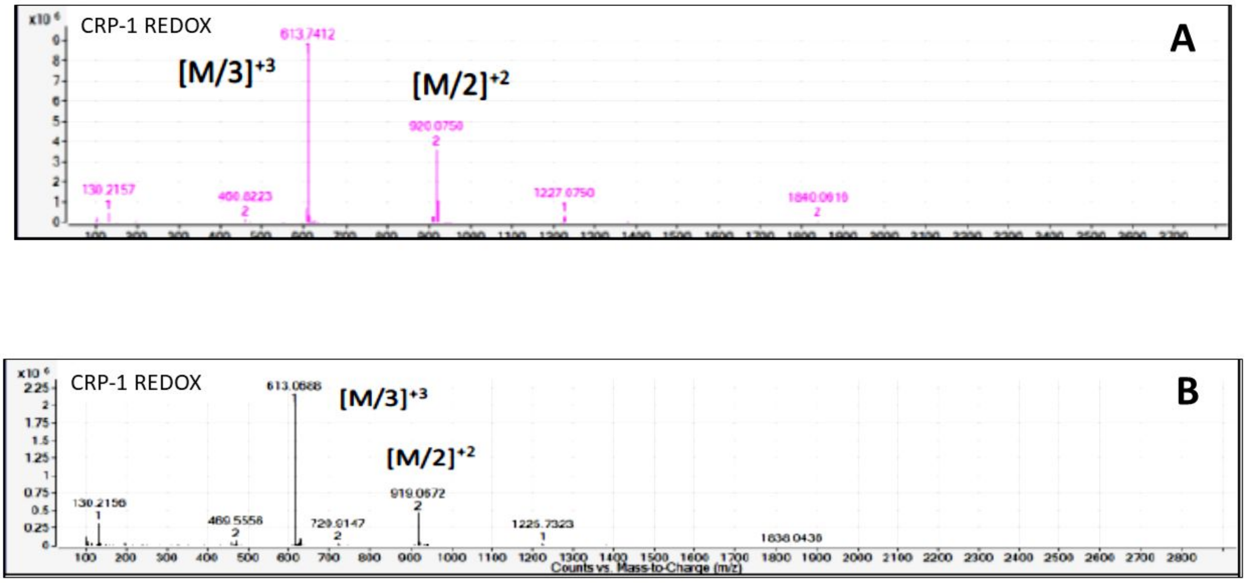

Figure S9: Mass spectrum of CRP-1 peptide: A) reduced version, B) oxidated compound.

Table S1. Amount of adsorbed moles on EDC/NHS treated surface.

[ ] peptide mg/mL	Abs bound	[ ]bound mg/mL	Adsorbed moles
0.25	0.016	0.18	53nmol
0.5	0.017	0.19	56nmol
1	0.033	0.43	126nmol
1.5	0.033	0.43	126nmol
2	0.033	0.43	126nmol

Table S2. Adsorbed moles on not treated surface.

[ ] peptide mg/mL	Abs bound	[ ]bound mg/mL	Adsorbed moles
0.25	0.006	0.036	10nmol
0.5	0.005	0.022	6.5nmol
1	0.01	0.09	26nmol
1.5	0.01	0.09	26nmol
2	0	0	0nmol
